# The biallelic novel pathogenic variants in *AGL* gene in a chinese patient with glycogen storage disease type III

**DOI:** 10.1186/s12887-022-03252-y

**Published:** 2022-05-16

**Authors:** Jing Wang, Yuping Yu, Chunquan Cai, Xiufang Zhi, Ying Zhang, Yu Zhao, Jianbo Shu

**Affiliations:** 1 Department of Gastroenterology, Tianjin Children’s Hospital, 300134 Tianjin, China; 2 Tianjin Children’s Hospital (Children’s Hospital of Tianjin University), 300134 Tianjin, China; 3 Graduate College of Tianjin Medical University, 300070 Tianjin, China; 4 Tianjin Pediatric Research Institute, 300134 Tianjin, China; 5 Tianjin Key Laboratory of Birth Defects for Prevention and Treatment, 300134 Tianjin, China; 6 Tianjin Pediatric Research Institute, Tianjin Children’s Hospital, No. 238 Longyan Road, Beichen District, 300134 Tianjin, China

**Keywords:** Glycogen storage disease type III, *AGL* gene, Frameshift variant, Novel variant, Whole-exome sequencing

## Abstract

**Background:**

Glycogen storage disease type III (GSD III) is a rare autosomal recessive glycogenolysis disorder due to *AGL* gene variants, characterized by hepatomegaly, fasting hypoglycemia, hyperlipidemia, elevated hepatic transaminases, growth retardation, progressive myopathy, and cardiomyopathy. However, it is not easy to make a definite diagnosis in early stage of disease only based on the clinical phenotype and imageology due to its clinical heterogeneity.

**Case presentation:**

We report a two-year-old girl with GSD III from a nonconsanguineous Chinese family, who presented with hepatomegaly, fasting hypoglycemia, hyperlipidemia, elevated levels of transaminases. Accordingly, Sanger sequencing, whole‑exome sequencing of family trios, and qRT-PCR was performed, which revealed that the patient carried the compound heterogeneous variants, a novel frameshift mutation c.597delG (p. Q199Hfs*2) and a novel large gene fragment deletion of the entire exon 13 in *AGL* gene. The deletion of *AGL* was inherited from the proband’s father and the c.597delG variant was from the mother.

**Conclusions:**

In this study, we identified two novel variants c.597delG (p. Q199Hfs*2) and deletion of the entire exon 13 in *AGL* in a Chinese GSD III patient. We extend the mutation spectrum of *AGL*. We suggest that high-throughput sequencing technology can detect and screen pathogenic variant, which is a scientific basis about genetic counseling and clinical diagnosis.

## Background

Glycogen storage disease type III(GSDIII; OMIM #232,400)also known as “limit dextrinosis”, “Cori” or “Forbes” disease, which was discovered by Barbara Illingworth and Gerty Cori1 in 1952, is a rare autosomal recessive glycogenolysis disorder with an incidence of 1:100,000 [1, 2]. Owing to the pathogenic variations in amyl glucosidase (*AGL*) gene (MIM #610,860), the glycogen debranching enzyme (GDE) is genetic deficiency or reduced activity, causing an accumulation of limit dextrin-like molecules in the cytoplasm of hepatocytes, myocytes, and other tissues [3].

**Fig. 1:**
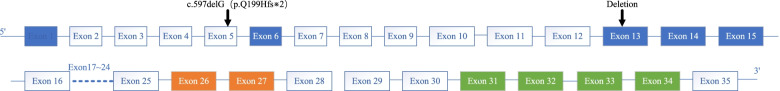
Schematic representation of the *AGL* gene comprising 35 exons. The variants in this study are written with black color. Blue boxes stand for exons considered encoding the putative transferase catalytic residues. Orange boxes stand for exons considered encoding the putative glucosidase catalytic residues and green ones stand for exons considered encoding putative glycogen-binding domain

The *AGL* gene has been isolated on chromosome 1p21 since 1991, and has been known as 85 kb in length with 35 exons encoding a 1532 amino acids protein(GDE) [4]. There are three important domains in *AGL*, the putative transferase catalytic residues, the putative glucosidase catalytic residues and the putative glycogen-binding domain (Fig. 1) [5]. Therefore, variants in these regions are more likely to influence the normal structure and function of *AGL*, resulting in the occurrence of GSD III. Due to alternative exon splicing and differential transcription of *AGL*, it has fix-transcript isoforms [6]. Isoform 1(NM_000642) is the major isoform, which consists of 34 exons and widely expresses in liver, muscle, kidney, and lymphoblastoid cells [7]. GDE is a 175-kD monomeric protein containing two independent catalytic domains, amylo-1-6-glucosidase (EC 3.2.1.33) and 1,4-α-D-glucan 4-α-D-glycosyltransferase (EC 2.4.1.25). It catalyzes one of the last steps in the conversion of glycogen to glucose-1-phosphate, playing an indispensable role in the glycogen degradation [5]. To date, over 180 different *AGL* gene pathogenic variants have been reported worldwide (Clinvar accessed in May 2021), reflecting a high degree of genetic heterogeneity. Among these variants, the majorities are nonsense variants followed by frameshift, splice site, and missense variants (https://www.ncbi.nlm.nih.gov/clinvar).

Individuals with GSD III are characterized by progressive myopathy and liver disease. Based on differences in tissue expression level of the GDE, there are four subtypes of GSD, including GSD IIIa, IIIb, IIIc and IIId [8, 9]. GSD IIIa is the most common subtype, accounting for about 85% [3]. It mainly affects liver, skeletal, and cardiac muscle. Patients with GSD IIIb, which affects only the liver and account for about 15% of all GSD III patients [10]. It has been confirmed that selective loss of glucosidase activity or transferase activity is the pathogenic mechanism of GSD IIIc and GSD IIId, respectively [11]. Both GSD IIIc and GSD IIId are extremely rare. GSD III is usually developing in infancy and early childhood, presenting as hepatomegaly, growth retardation, fasting hypoglycemia, hyperlipidemia, elevated hepatic transaminases, progressive myopathy and cardiomyopathy [12, 13]. The hepatomegaly may disappear in adolescence and adulthood. Meanwhile, in patients without cirrhosis or myopathy, all symptoms usually improve during adolescence [14]. Muscle weakness is a slow progress, typically becoming prominent in the third to fourth decade [15]. Another common associated symptom is hypertrophic cardiomyopathy, which usually develops between most of the children with GSD IIIa. The clinical presentation is heterogeneous, ranging from asymptomatic to severe cardiac dysfunction, such as congestive heart failure and sudden death [16].

Here, we reported a patient whose clinical diagnosis was considered as glycogen storage disease III. Whole exome sequencing (WES), Sanger sequencing, and quantitative real time polymerase chain reaction (qRT-PCR) was performed on the proband and her parents to make an accurate diagnosis. Our findings provide a wider range of *AGL* gene variants spectrum in patients with GSD III in the Chinese population.

## Case presentation

**Fig. 2:**
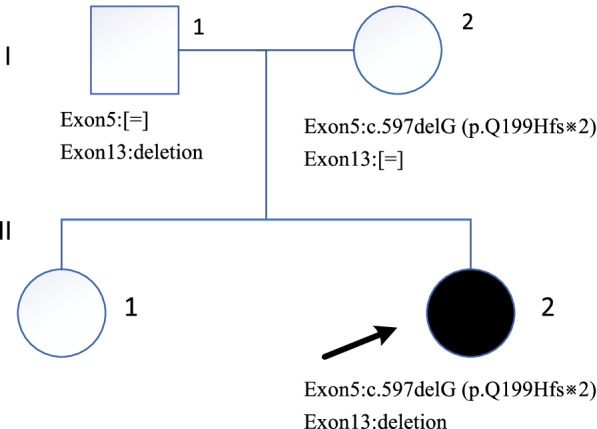
Pedigree of the GSD III family with the variants of exon 5 and exon 13. Here, the circles indicate female individuals and rectangles indicate males. The patient of the study is indicated by a black circle. The patient (II-2) contains two variations in *AGL* gene, the c.597delG(p.Q199Hfs※2)in exon 5 is inherited from the mother (I-2) and the deletion of exon 13 is inherited from the father(I-1)

A two-old-girl was admitted to Tianjin Children’s Hospital due to progressive enlargement of the abdomen resulting from hepatomegaly. The parents of the patient are non-consanguineous. The pedigree of the family is depicted in Fig. 2. Laboratory examinations revealed nocturnal fasting hypoglycemia, hyperlipidemia, significantly elevated levels of transaminases. Hepatomegaly was confirmed with abdominal ultrasound and abdominal computed tomography enhanced scan. These clinical phenotypes suggested GSD III. However, electromyography excluded myopathy, cardiomyopathy was ruled out by electrocardiogram and echocardiography, and physical examination showed no obvious growth retardation.

Biochemical, imaging, clinical and diet therapy data at baseline, 8 days and 20 days are shown in Table 1.

**Table 1:** Biochemical, imaging, clinical and diet therapy data of the patient

	Before	8 days later	20 days later
*Clinical data*			
Age (years)	2	2	2
Height (cm)	85	85	85
Weight (kg) Abdominal circumference(cm) Right subcostal liver(cm)	14.2	14.2	14.2
	59.5	60.0	59.0
	10	10	10
*Biochemical data*			
Pre-prandial fasting blood glucose (mmol/L)	4.64	5.67	6.1
Nocturnal blood glucose(mmol/L)	-	1.3	5.6
Morning fasting blood glucose (mmol/L)	2.5	4.1	6.5
Lactate (normal range 0.5–2.22mmol/L)	5.54	-	-
Triglycerides (normal range 0–2.26 mmol/L)	5.45	3.71	2.4
Cholesterol (normal range 0–5.2 mmol/L)	5.50	4.96	5.05
Creatine kinase (normal range 40–200 U/L)	213	141	-
Aspartate transaminase (normal range 13–35 U/L)	1154.6	452	532
Alanine transaminase (normal range 7–40 U/L)	781.6	282	165
γ-glutamyl transpeptidase (normal range 7–45 U/L)	98	101	84
Troponin T (normal range 0–0.0223 ng/mL)	21.0	-	-
Myoglobin (normal range 25–58 ng/mL)	0.01	-	-
*Imaging data*			
Abdominal ultrasound	Hepatomegaly	-	Hepatomegaly Below umbilical level 55 mm
Abdominal computed tomography enhanced scan	Hepatomegaly No obvious abnormal enhancement	-	-
Liver or muscle biopsy	-	-	-
Electromyography	normal	-	-
Electrocardiogram	normal	-	-
Echocardiography	normal	-	-
Chest computed tomography	normal	-	-
Head computed tomography plain scan	-	-	-
Skeletal system x-rays	-	-	-
*Diet therapy*	Regular diet	Start uncooked cornstarch treatment 1-2 g/kg/d	Adhere to uncooked cornstarch treatment 1-2 g/kg/d

‘-‘means no done

**Fig. 3:**
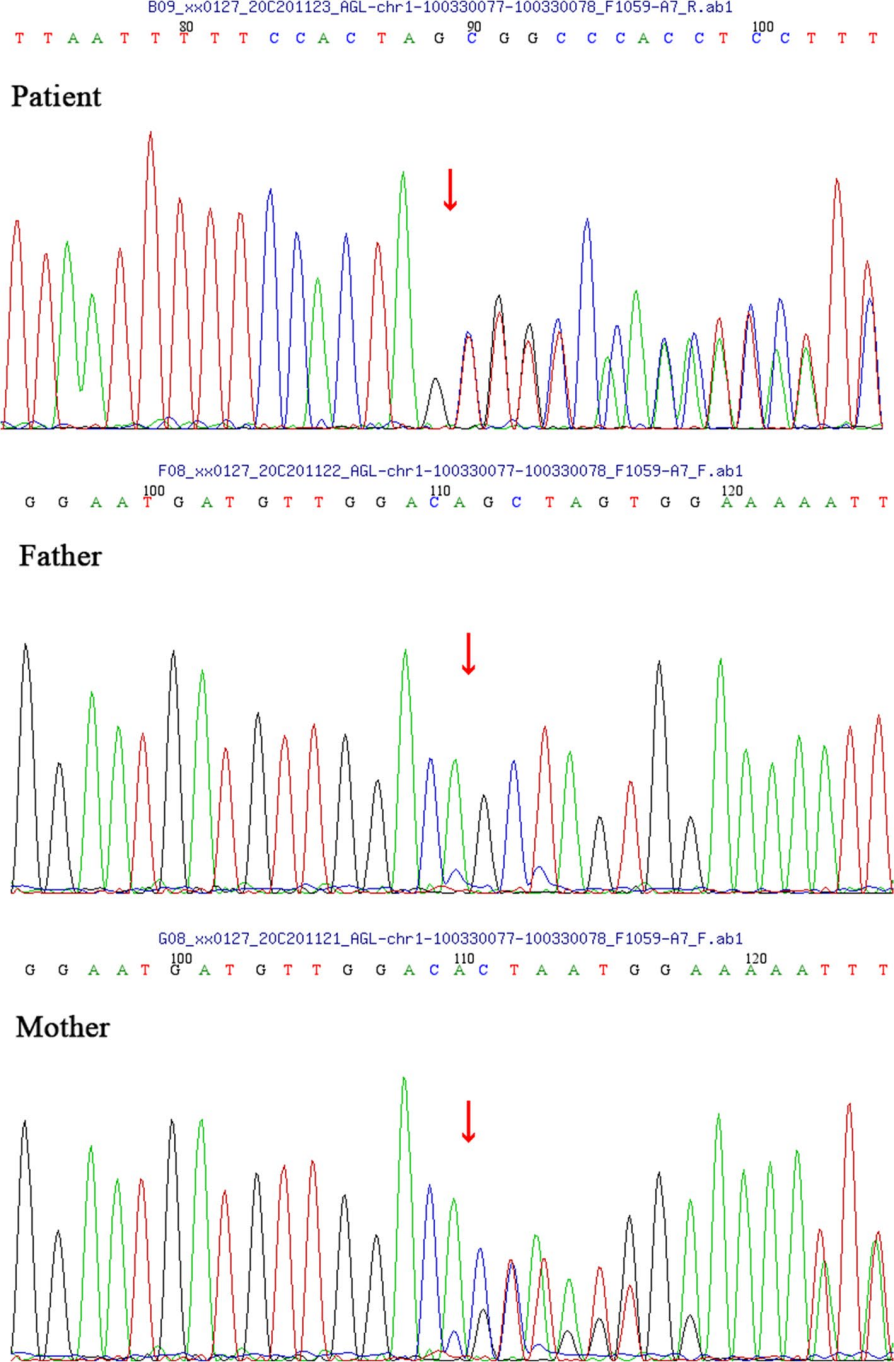
Sanger sequencing of the patient and her parents. The figure shows the frameshift variant c.597delG and indicates that the variant was inherited from the proband’s mother. The red arrow shows the site of variant

**Fig. 4:**
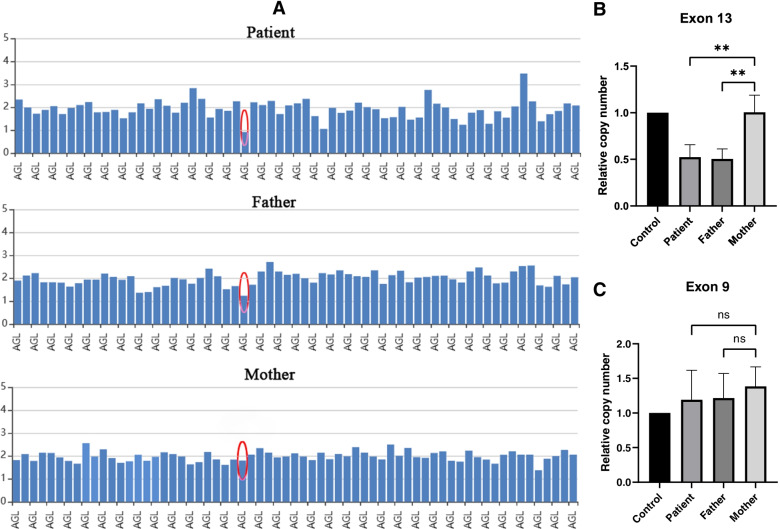
Gene deletion and duplication analysis map. **A** Multiplex Ligation-dependent Probe Amplification (MLPA) of the patient and her parents; **B** shows qRT-PCR results of exon 13 in *AGL* gene: the relative copy number in the control and the patient’s mother was normal, and the proband and his father were half of the normal; **C** shows qRT-PCR results of exon 9: the relative copy number in four groups were normal. All these indicates the proband has a heterozygous deletion in *AGL* gene (exon13), and shows the deletion was inherited from the proband’s father. The Red circle shows the region of fragment deletion, ** means *P* < 0.01, ‘ns’ means *P* > 0.05

To make a definite diagnosis, the peripheral blood of the patient and parents were sent to MyGenostics company (Beijing, China) for whole-exome sequencing (WES), Sanger sequencing, and qRT-PCR. Genetic analysis revealed a biallelic novel pathogenic variants in *AGL* gene. Sanger sequencing identified a previously unreported heterozygous variant in exon 5 of the *AGL* gene: c.597delG (p. Q199Hfs*2) (NM_000642), which inherited from the mother (I-2) (Fig. 3). This variant causes the glutamine at position 199 is changed to histidine, leading to the insertion of a stop codon at position 200. Furthermore, the heterozygous deletion of exon 13 found by WES was also a previously unreported variant, which inherited from the father(I-1). The results of qRT-PCR showed that the relative copy number of exon 13 in the control and the patient’s mother were normal, while the proband and his father were half of the normal, confirming the absence of exon 13 (Fig. 4). The deletion of exon 13 causes deletions of amino acid from position 538 to 578, leading to the termination transcription at the 540th amino acid. Both variants could cause the early termination of gene transcription. The patient (II-2) inherited both variants and was therefore a compound heterozygote. The sister (II-1) had no symptoms of GSD III, who did not performed WES.

## Discussion and conclusions

In our study, we reported a two-year-old girl (Fig. 2, II-2) from the region of China. She came to hospital for treatment mainly because of hepatomegaly. All examinations performed that she had hepatomegaly, nocturnal fasting hypoglycemia, hyperlipidemia, significantly elevated levels of transaminases, and midface hypoplasia with a depressed nasal bridge. However, she did not have any symptoms or exam evidence of myopathy or cardiomyopathy.

The cardinal clinical features of GSD III are hepatomegaly, fasting hypoglycemia, elevated serum concentrations of transaminases and CK [17]. The diagnosis is established by identification of biallelic pathogenic variants in *AGL* gene using molecular genetic testing such as single-gene testing or whole exome sequencing [18]. Liver or muscle biopsy can certify deficient glycogen debranching enzyme activity and accumulation of limit dextrin-like molecules in either liver or muscle biopsy specimen, so they are always used to distinguish different subtypes [19]. However, biopsy is more invasiveness and not required to make the diagnosis of GSD III, it is rarely performed. In our case, due to the typical clinical symptoms, the patient shown high clinical suspicion of GSD III. To make clear diagnosis, we performed genetic analysis technology on the proband and her parents. The result laid out that the proband had a biallelic likely pathogenicity variants in *AGL* gene. Thus, the patient was diagnosed as GSD III.

Unfortunately, due to the invasive of biopsy the patient’s family disagreed with the operation, and no further detection of liver or muscle branch enzyme activity was carried out. Hence, the clinical subtype of the patient could not be confirmed. Since there was no obvious growth retardation in the patient, no further evaluation was undertaken, such as imaging examination of the skeletal system, etc. Therefore, follow-up in the later stage is very necessary.

To date, there are over 180 different *AGL* gene pathogenic variants registered in the Clinvar database (https://www.ncbi.nlm.nih.gov/clinvar). It includes all variant types: missense, nonsense, splice site, frameshift, and large gene fragment deletions and duplications. The majorities are frameshift (39.0%), nonsense (36.2%), and splice-site (10.4%) variants. Depending on ethnic groups, the prevalent mutations of *AGL* vary in GSD III. Information on the spectrum of *AGL* variants will improve molecular diagnosis of GSD III in these populations and genetic counseling. For example, the most frequent variant among Italian patients is splice-site variant (c.2681 + 1G > A), the common nonsense variant in the Faroe Islands is p.R408X, and the prevalent variants in the United States are p.R864X and c.3964delT [20]. In Asian, c.1735 + 1G > T shows the highest frequency in Japanese, Korean and Chinese patients [20]. The common variant in Turkish patients is p. W1327X [21]. Despite these, there is still high heterogeneity of *AGL* variants worldwide and pathogenic variants scattered throughout the gene [22]. In our case, the patient carries two compound-heterozygous variants (c.597delG and deletion of exon 13) in *AGL*. According to the ACMG standards and guidelines for the interpretation of sequence variants [23], the maternal frameshift variant c.597delG is a 1 bp deletion encompassed in exon 5, which causes a shift in the open reading frame and insertion of a premature termination codon (p. Q199Hfs*2), resulting in a truncating protein. This will lead to the loss of protein function and the occurrence of GSD III (PVS1). The variant is absent from controls in Exome Sequencing Project, 1000 Genomes or ExAC (PM2). Therefore, variant c.597delG is considered a likely pathogenic variant. Otherwise, the large gene fragment deletion in *AGL* gene with a paternal origin is a 124 bp deletion of the entire exon 13. It results in a premature truncation of the protein, leading to the loss of protein function (PVS1). The variant is absent from controls in Exome Sequencing Project, 1000 Genomes or ExAC (PM2). Thus, variant deletion of exon 13 is classified as likely pathogenic variant of *AGL*. Those two variants are novel and no registered in the Clinvar or the HGMD. The patient was diagnosed with GSD III according to the typical clinical features of liver disease hepatomegaly, nocturnal fasting hypoglycemia, hyperlipidemia, significantly elevated levels of transaminases. Those are consistent with the result of genetic analysis a biallelic novel pathogenic variants in *AGL* gene.

There is poor correlation between the genotype and phenotype of GSD III. Moderate to severe disease (GSD IIIa) associated with homozygosity for loss of function variants [24]. Frameshift, nonsense, and splices site variants are possible significant association with elevated CK and TC, suggesting that these variants were associated with severe phenotype [5, 20]. To evaluate genotype–phenotype relationships in GSD III with similar milder phenotype, we compared clinical features among patients with *AGL*-variants published to date (Table 2). In earlier reports, variants of exon 3, such as c.16 C > T and c.18_19delGA, along with a second mutation elsewhere in the *AGL* gene were reported to be associated with GSD IIIb [9]. This is consistent with our observation [24–27]. Otherwise, we observed milder phenotype has been found to be associated with IVS32–12 A > G homozygosity variant in intron 32 of the *AGL* gene [28, 29]. As is evident from Table 2, typical clinical features of mild GSD III are liver disease hepatomegaly, fasting hypoglycemia, hyperlipidemia, significantly elevated levels of transaminases, with or without elevated serum concentrations of CK. The increased serum CK is a suggestive and nonspecific marker of muscle breakdown. There are correlations between premature termination codon (PTC) variants and elevated serum CK value. Otherwise, the missense and small in-frame deletion mutations are associated with a normal serum CK level in GSD III [20]. However skeletal muscle and cardiologic complications were usually minimal during childhood, progressive myopathy can develop after the third or fourth decade of life [30]. In our study, the patient manifests as liver damage, and has no evidence for myopathy or cardiomyopathy up to now, which may associate with the age at diagnosis, disease classification or heterozygous variants of *AGL*. We considerate the patient probably GSD IIIb (though not confirmed) as clinical and biochemical data of the patient presented here rule out muscular manifestations. So, if there is no muscle damage in the patient during the late follow-up or confirmed by muscle biopsy as GSD IIIb, our report and the previous report [29] provide evidence that variants in exons other than exon 3 could be responsible for the GSD IIIb phenotype. Hyperlipidemia may relate to frameshift variant (c.597delG). Although previous reports suggest that PTC is associated with elevated levels of serum CK, our study shows a normal serum CK level, which may due to the patient’s age or measurement in resting state [7]. These differences indicate the vary widely heterogeneity of phenotype. The follow-up date and further research is needed to clarify the relationship between PTC and CK. It also helps to understand the disease development and guide treatment in the future.

**Table 2:** Spectrum of *AGL*-variants and clinical features in patients with milder phenotype of GSD III

Reference	Age/sex	Ethnicity or origin of study	Hepatomegaly	Liver biopsy	Cardio myopathy	Myopathy	CK	Hypertriglyceridemia	AST, ALT	Hypoglycemia	Sub type	AGL mutation
Crushell et al. 2010 [24]	18Y	Irish	+	N/A	-	-	-	N/A	+	+	IIIb	c.[1879G>T]+[4197delA] exon 15/exon 32
	18Y		+	N/A	-	-	-	N/A	+	+	IIIb	c.[18_19delGA]+[3682C>T] exon 3/exon 28
	20Y		+	N/A	-	-	-	N/A	+	+	IIIb	c.[18_19delGA]+[3980G>A] exon 3/exon 31
	17Y		+	N/A	-	-	-	N/A	+	+	IIIb	c.[18_19delGA]+[3980G>A] exon 3/exon 31
Lee et al. 2011 [25]	16Y/F	Nicaraguan	+	+	-	-	-	N/A	+	+	IIIb	17delAG/ Hom
Mili et al. 2012 [28]	2Y/M	Tunisian	+	N/A	N/A	-	-	N/A	+	+	IIIb	IVS32–12A>G/ Hom Intron 32
Minen et al. 2012 [31]	3Y/M	Italian	+	+	-	-	-	+	+	+	N/A	c.664+3A>G/ Hom Intron 6
Ben et al. 2013 [32]	11M/M/ Con	Tunisian	+	+	+	-	-	+	+	+	N/A	c.2682-8A>G/ Hom Intron 21
	14D/F/ Con		+	+	-	-	-	+	+	+	N/A	
Sentner et al. 2013 [26]	3Y/M	Caucasian	+	+	-	-	-	+	+	+	N/A	c.[655A>G]+ [4529dupA] exon 6/exon 35
	30Y/M		+	+	-	-	-	+	+	+	IIIb	c.16C>T/ Hom exon 3
	41Y/F		+	+	-	-	-	+	+	+	IIIb	c.1027C>T+ IVS32–12A>G exon 9/Intron32
	3Y/M/ Con		+	+	-	-	-	+	+	+	N/A	c.3911delA/ Hom exon 30
	1Y/M/ Con		+	+	-	-	+	+	+	+	N/A	
Ko et al. 2014 [30]	5Y/F	Korean	+	+	-	-	-	-	+	N/A	N/A	p.[R285X]+[R675W]
	12Y/M		+	+	-	-	-	-	+	N/A	N/A	c.[1735+1G>T]+ [2591G>C]
Basit et al. 2014 [29]	Con A 5Y/M 9Y/F 11Y/ F	Saudi	+	N/A	-	-	+	N/A	+	+	IIIb	VS32-12A>G/ Hom Intron 32
			+	N/A	-	-	+	-	+	+	IIIb	
			+	N/A	-	-	N/A	N/A	+	+	IIIb	
	Con B 12Y/M 8Y/M	Saudi	+	N/A	-	-	N/A	N/A	+	-	IIIb	No detect any pathogenic variant
			+	N/A	-	-	+	N/A	+	-	IIIb	
	Con C 2Y/F	Saudi	+	N/A	-	-	-	N/A	+	-	IIIb	VS32-12A>G/ Hom Intron 32
	Con D 36Y/ F 42Y/ F 44Y/M 44Y/ F 46Y/ F 50Y/M 11Y/ F	Saudi	+	N/A	-	-	N/A	N/A	N/A	+	IIIb	VS32-12A>G/ Hom Intron 32
			+	N/A	-	-	+	N/A	+	+	IIIb	
			+	N/A	-	-	N/A	N/A	N/A	+	IIIb	
			+	N/A	-	-	N/A	N/A	N/A	+	IIIb	
			+	N/A	-	-	N/A	N/A	N/A	+	IIIb	
			+	N/A	-	-	+	N/A	+	+	IIIb	
			+	N/A	-	-	+	N/A	+	+	IIIb	
	Con E 4Y/ F 11Y /F	Saudi	+	N/A	-	-	N/A	N/A	N/A	+	IIIb	VS32-12A>G/ Hom Intron 32
			+	N/A	-	-	+	N/A	+	+	IIIb	
Oterdoom et al. 2015 [27]	63Y/M	Dutch	+	+	-	-	-	-	+	-	IIIb	c.[16C>T]+[1013_1014dupAT]
Perveen et al. 2020 [5]	3Y/M	Indian	+	N/A	N/A	N/A	-	-	+	-	N/A	c.1880A>G/ Hom
	3Y/F		+	+	N/A	N/A	-	+	+	-	N/A	
	4Y/F		+	N/A	N/A	N/A	-	-	+	+	N/A	c.4331A>G/ Het
	3Y/M		+	+	N/A	N/A	-	-	+	+	N/A	c.3069G>A/ COH
	6Y/F		+	+	N/A	N/A	-	-	+	-	N/A	c.4353G>T/ COH
	2.5Y/F		+	+	N/A	N/A	-	+	+	+	N/A	c.3083+1G>A/Het
	3.5Y/M		+	+	N/A	N/A	-	+	+	-	N/A	c.2362-2392dup31/ COH
	4Y/M		+	+	N/A	N/A	-	+	+	+	N/A	c.[4334A>G]+[3444C>G]
	3Y/M		+	+	N/A	N/A	-	+	+	-	N/A	c.[2362-2392dup31]+[4334A>G]

*Abbreviations: AGL *amylo-1,6-glucosidase, *D *day, *M* month, *Y* year, */F *female, /*M* male, *CK* creatine kinase, *ALT* alanine aminotransferase, *AST* aspartate aminotransferase; *Hom* homozygote, *Het* heterozygote, *COH* Compound heterozygote, *Con* consanguineous, *N/A* not available/assessed

The treatment of GSD III is still controversial, and the recognized mainstay treatment is dietary management to maintain euglycemia, including uncooked cornstarch therapy with a nocturnal high-protein supplementation [33]. Recently, high fat diet has been confirmed a benefit effect to the progress of cardiomyopathy and myopathy [34]. In the past few years, significant progress has been made in gene therapy, which is expected to be more effective in the future for the treatment with GSD III [35]. In this research, the patient was treated with uncooked cornstarch 1-2 g/kg/d after being diagnosed with GSD III 8 days later. After a period of dietary control, her nocturnal fasting blood glucose returned to normal. 20 days after admission, compared to the situation at the time of admission, there was no retraction in the size of the liver by abdominal ultrasonography, but the transaminase decreased.

In summary, we identified two novel variants c.597delG (p. Q199Hfs*2) and deletion of the entire exon 13 in AGL in a Chinese GSD III patient. We extend the mutation spectrum of AGL in Chinese patients with GSD III and point out to the heterogenous genetic background of the disease, providing theoretical guidance for genetic counseling and precision therapy. Our results suggest the need for complete AGL gene sequencing to diagnose the disease based on a suggestive clinical feature and provide genetic counseling for young families. However, it is limited to hypothesize on the correlation between genotype and phenotype just based on this patient only. In the future, we will continue to collect cases for cohort studies in order to expand the basic information of the disease and establish genotype-phenotype correlation.

## Data Availability

The raw datasets used and analysed during the current study are not deposited in publicly available repositories because of considerations about the privacy or security of human. However, the datasets can be available from the corresponding author on reasonable request.

## Abbreviations

*ALG*: Amyl glucosidase gene
GSDIII: Glycogen storage disease type III
GDE: Glycogen debranching enzyme
WES: Whole exome sequencing
ALT: Alanine aminotransferase
AST: Aspartate aminotransferase
CK: Creatine kinase
PTC: Premature termination codon
